# Associations of Mercury Exposure with Serum Sex Steroid Hormones in Children 6-18 Years from NHANES 2013-2016

**DOI:** 10.2174/0118715303396515250912001734

**Published:** 2025-09-25

**Authors:** Binwei Qiu, Yan Lin

**Affiliations:** 1 Department of Pediatrics, Longyan First Affiliated Hospital of Fujian Medical University, Longyan 364000, Fujian, China

**Keywords:** Mercury exposure, sex steroid hormones, children, sex difference, puberty status, public health, testosterone

## Abstract

**Introduction:**

To examine the relationship between mercury exposure and serum sex steroid hormones among children aged 6-18 years.

**Methods:**

Data were collected from the NHANES 2013-2016. A cohort of 2,637 Children with available information on mercury exposure, serum sex steroid hormones, and covariates was enrolled. Generalized linear models were applied to explore the association of mercury exposure with sex steroid hormones. Finally, we adopted the Bayesian kernel machine regression (BKMR) model to investigate the effect of mixture exposure to mercury on hormone levels.

**Results:**

Methyl mercury (MeHg) and total mercury (Hg) were associated with all sex steroid hormones except E2 (*P* <0.05). After adjusting for covariates, MeHg and Hg were related to sex hormone-binding globulin (SHBG) and free androgen index (FAI) (*P* <0.05). Stratified analysis by sex-puberty revealed a negative relationship between Hg and SHBG, while a positive association of MeHg with FAI in prepubertal girls (*P* <0.05). Their associations were confirmed by the BKMR model analysis, in which the mixture exposure was positively linked to FAI but inversely related to SHBG, particularly among prepubertal girls.

**Discussion:**

These findings suggest that mercury may disrupt sex hormone homeostasis during critical developmental windows. Appropriate measures should be implemented to mitigate the adverse effects of mercury on sex hormones in the young population. However, future longitudinal large-scale studies are required for validation.

**Conclusion:**

Exposure to MeHg and Hg is associated with increased FAI levels, but decreased levels of SHBG, which are more pronounced in girls. The heightened susceptibility of prepubertal girls underscores the need for targeted public health interventions, including dietary guidance on seafood consumption and early biomonitoring of mercury levels in high-risk populations, to mitigate potential health impacts.

## 1 INTRODUCTION

Sex steroid hormones are essential for the development and function of the reproductive system during childhood and adolescence [1-3]. Testosterone (TT) and estradiol (E2), play a crucial role in the development and functioning of the reproductive system [4-10]. Sex hormone-binding globulin (SHBG), a protein found in plasma, acts as a carrier molecule, binding and transporting TT and E2, thereby regulating the levels of biologically active, non-protein-bound sex hormones [4, 11-13]. TT and its metabolites exert widespread and essential effects in shaping the secondary sexual characteristics of adolescent boys [14, 15]. The majority of TT in the bloodstream is bound to SHBG, serving as the primary form for its functional role [16]. Furthermore, while E2 primarily governs female sexual development, it also plays a role in male fertility [17].

As industrialization progresses, heavy metal ions are inevitably emitted into the agricultural lands and freshwater environments, leading to increased human intake through dietary sources [18-20]. Among the heavy metals, mercury is notorious for its extreme toxicity [21-23]. Natural mercury exists in three forms: metallic mercury, organic mercury, and inorganic mercury [24, 25]. Methyl mercury (MeHg), an organic form, is the most prevalent and toxic variant [26, 27]. Prior studies have already established the disruptive impact of MeHg on the reproductive endocrine system [28]. Tartu *et al.* suggested that the adverse effects of mercury on the reproductive system are influenced by age [29]. Furthermore, the detrimental effect of mercury on reproductive hormone levels in women has also been documented [30]. However, the correlation between mercury and serum sex hormones in the younger population remains elucidated.

Hence, the objective of this study was to employ multiple statistical approaches, including univariable and multivariable linear regression models, as well as Bayesian kernel machine regression (BKMR), to comprehensively examine the associations between mercury and sex steroid hormones in children under the age of 18 using the U.S. National Health and Nutrition Examination Study (NHANES) database. Additionally, we sought to investigate whether the associations were modified by puberty-gender status.

## 2 MATERIALS AND METHODS

### 2.1 Data Source and Sample

The analytic sample was obtained using the NHANES database, which examines a nationally representative sample of about 5,000 persons each year. The survey conducted by the National Center for Health Statistics is unique in that it combines interviews and physical examinations. The computer-assisted Personal Interview system contains consistency checks to reduce data entry errors and uses online help screens to assist interviewers in defining terms in the questionnaire. Informed consent was acquired from all the participants, and study protocols were approved by the NCHS Ethics Review Board.

G*Power software (version 3.1.9.7) was used to estimate the sample size by inputting the following parameters: the statistical test was two-tailed, effect size = 0.5, the significance level was set at α = 0.05, and power was set to 0.95. This yielded a minimal sample size of 220. Herein, we downloaded data from NHANES cycles 2013-2014 and 2015-2016 with a total of 19,337 samples. Children are persons aged or less than 18 according to the United Nations Convention on the Rights of the Child [United Nations Youth. [(accessed on 1 January 2020)];2020 Available online: https://www.un.org/en/sections/issues-depth/youth-0/index.html]. Inclusion criteria: aged 3-18 years (n=6,445); with available information on sex hormone measurements (n=3,578). Exclusion criteria: with a history of using sex hormone medication (n=0); missing mercury data (n=941). This study included a final sample of 2,637, meeting the required sample size.

### 2.2 Exposure Variable

The blood concentration of inorganic mercury (InHg), ethyl mercury (EtHg), MeHg, and total mercury (Hg) were considered as exposure variables. The quantification of InHg, EtHg, and MeHg in whole blood samples is performed using a triple spike isotope dilution method employing gas chromatography to separate the species, followed by introduction into an ICP-DRC-MS for detection. The Hg content of blood specimens was measured using mass spectrometry after a simple dilution and sample preparation step. All the results match the Division of Laboratory Science’s quality control and quality assurance performance standards for accuracy and precision, similar to the Westgard rule [31]. A detailed description of laboratory methodology can be accessed on the official website (https://www.cdc.gov/nchs/nhanes/index.htm).

For analytes with results below the lower limit of detection (LOD), the value is the LOD divided by the square root of 2, as recommended by NHANES [32]. The LODs for InHg, EtHg, MeHg, and Hg were 0.21 ug/L, 0.064 ug/L, 0.26 ug/L, and 0.28 ug/L, respectively.

### 2.3 Outcome Variable

The serum sex steroid hormone indicators of total TT, E2, and SHBG served as outcome variables. Serum samples were processed and stored at −20 °C until they were shipped to the National Center for Environmental Health for testing. TT and E2 in serum were measured using the isotope chromatography-tandem mass spectrometry method, which demonstrates high accuracy and precision. SHBG measurement was based on the reaction of SHBG with immune-antibodies and chemiluminescence measurements of the reaction products that occur after two incubation periods and are subjected to a magnetic field. The LODs for TT, E2, and SHBG were 12.44 ng/dL, 22.21 pg/mL, and 52.07 nmol/L, respectively. Besides, we calculated the free androgen index (FAI) by the ratio of TT to SHBG. The ratio of TT to E2 (TT/E2) was also calculated. FAI and TT/E2 had an indirect assessment of the approximate amount of circulating free TT [33, 34].

### 2.4 Covariates

Covariates were identified following the previous reports regarding the association between chemical exposure and sex hormone homeostasis [35]. Age, gender (girls and boys), body mass index (BMI), race (Hispanic, non-Hispanic White, non-Hispanic Black, and other races), education level (categorized by less than 9th grade and more than 9th grade), poverty income ratio (PIR), and physical activity information were obtained by questionnaires. Shellfish/fish eaten during the past 30 days were collected from the dietary interview. Cotinine exposure status was divided into the unexposed group (LOD <0.015) and the exposed group (LOD ≥0.015). In addition, the time of blood sampling (morning, afternoon, and evening) and a months (November 1 through April 30 and May 1 through October 31) were collected to account for the effects of time fluctuations on sex steroid hormones.

### 2.5 Statistical Analysis

We conducted descriptive statistics to describe the population characteristics by gender groups. The *Chi-square* test and the *Manny-Whitney U* test were employed to compare group differences for the categorical variable and continuous variable, respectively. The distribution of the sex steroid hormones and chemical exposure levels was described using the mean and selected percentiles (25^th^, 50^th^, and 75^th^). Due to the same value of InHg and EtHg at three percentiles, they were excluded from the following analyses.

Next, the generalized linear models (GLM) were adopted to assess the association of MeHg and Hg as a continuous variable with sex steroid hormone levels among all children. This association was further analyzed by categorizing MeHg and Hg in quartiles. Tests for linear trends were conducted by using the median value for each quartile and fitted as a continuous variable in the regression models. In the univariable model, no covariate was adjusted. Age, gender, race, BMI, education level, physical activity, PIR, cotinine exposure, time of blood sampling, and six month time period were adjusted in the multivariable model. Moreover, we examined the association of MeHg and Hg with sex steroid hormones in boys and girls according to puberty status: pubertal group (TT ≥50 ng/dL for boys or E2 ≥20 pg/mL for girls) and prepubertal group (TT <50 ng/dL for boys or E2 <20 pg/mL for girls) [36].

The BKMR model allows more flexible models of the overall joint effect of the mixture as well as individual estimates of exposure-outcome associations [37]. We performed BKMR to analyze the single and mixed exposure using the R package bkmr, and conducted 1,000 iterations with all covariates adjusted. The function “OverallRiskSummeries” was used to visualize the overall risk effects of the mixtures, calculated by comparing the change in sex hormones when two predictors were at a particular percentile to when the two predictors were at the 50th percentile. Next, the univariate exposure-response function was explored when a single chemical was at the 75th percentile compared to the 25th percentile, while the other chemical was at the 50th percentile. The two predictors were included using the variable selection option to assess the individual posterior inclusion probability (PIPs) and identify the relative importance of individual exposure variables to the overall mixture effect.

All statistical analyses were performed using SPSS software version 23.0 and R Studio version 4.2.2). *P-value* less than 0.05 was considered statistically significant.

## 3 RESULTS

### 3.1 Characteristics of the Participants

The demographic characteristics of the study population are shown in Table **1** and Figs. (**1A**-**1E**). A total of 2,637 samples were enrolled (1,317 boys and 1,320 girls) with a median age of 10 years and a median BMI of 19.6 kg/m^2^. Hispanic, non-Hispanic White, non-Hispanic Black, and other races accounted for 36.822%, 26.849%, 21.578%, and 14.752%, respectively. Major participants were in less than the 9th grade and exposed to cotinine. The median PIR was 1.490. Blood sampling in the morning, afternoon, and evening took a proportion of 41.904%, 36.860%, and 21.236%, respectively. The proportion of six months time period on November 1 through April 30 and May 1 through October 31 was 47.327% *versus* 52.673%. There were 51.688% of cases that had eaten shellfish/fish during the past 30 days. Except for BMI and cotinine exposure (*P* <0.05), there were no significant differences in other parameters, including age, race, education level, PIR, physical activity, time of blood sampling, six months’ time period, and shellfish/fish eaten during the past 30 days between boys and girls (all *P* >0.05). Table **2** exhibited the distribution of the sex hormone levels and mercury levels, including the mean value, 25th, 50th, and 75th percentiles. Since the value of InHg was 0.19 at three percentiles and the value of EtHg was 0.11 at three percentiles without change, InHg and EtHg were removed in the following analyses.

### 3.2 Association of MeHg and Hg With Sex Steroid Hormone Levels

In the univariable analysis, MeHg as a continuous variable was positively related to TT, FAI, and TT/E2, but negatively associated with SHBG (*P* <0.05). No significant relationship between MeHg, Hg, and E2 was observed (*P* >0.05) (Fig. **2**). Taking MeHg Q1 as a reference, β of the association of MeHg with TT, SHBG, FAI, and TT/E2 in Q4 was 34.272, -9.811, 130.447, and 1.608, respectively. Similarly, there was a positive association of Hg with TT, FAI, and TT/E2, while a negative relationship between Hg and SHBG was found (all *P* <0.05). In terms of the association of Hg with sex hormone levels TT, SHBG, FAI, and TT/E2, β in the Q4 group was 33.012, -9.934, 133.451, and 1.606, respectively, compared with the Q1 group (all *P* <0.05). Moreover, the TT, FAI, and TT/E2 levels of participants showed a gradual upward trend with each increase of one grade of both MeHg and Hg levels (*P* for trend <0.05) (Fig. **3**).

After adjusting for age, gender, race, BMI, education level, physical activity, PIR, cotinine exposure, time of blood sampling, and six months’ time period, MeHg and Hg as a continuous variable was related to SHBG and FAI (*P* <0.05) but had no significant association with TT, E2, and TT/E2 (Fig. **2**). Whereas, MeHg and Hg as a categorical variable only had an intimate relation with SHBG (*P* <0.05). The test for linear trend showed that the levels of SHBG would decrease and FAI would increase with the increase of MeHg level (*P* for trend <0.05). A similar trend was observed in the association of Hg level with SHBG level and FAI level (Fig. **4**). The above findings suggested that MeHg and Hg were closely related to SHBG and FAI.

### 3.3 MeHg and Hg were Closely Related to FAI and SHBG in Prepubertal Girls

To investigate the modification role of gender-puberty on the association of MeHg and Hg with sex hormones, we conducted a multivariable GLM analysis. Compared with puberty status, the relationships between chemical exposure and sex hormones were more pronounced among prepubertal girls. Specifically, there was no significant association of MeHg and Hg with any sex hormone indicator regardless of the pubertal boys or girls (all *P* >0.05). Although MeHg and Hg had no obvious relation with TT, E2, and TT/E2 among prepubertal children, MeHg was positively linked to FAI, and Hg was inversely related to SHBG in prepubertal girls (*P* <0.05) (Table **3**). When taking shellfish/fish eaten during the past 30 days into consideration, MeHs was still positively associated with FAI in prepubertal girls (*P* <0.05) (Table **S1**). These results indicated the important association of MeHg and Hg with FAI and SHBG, especially in prepubertal girls.

### 3.4 The association of MeHg and Hg with FAI and SHBG by the BKMR Model

The BKMR results for estimating the overall effect and the exposure-response functions of the mixture of two chemicals on sex hormones are exhibited in Fig. (**5**). The chemical mixture was positively related to FAI but had a negative association with SHBG in the BKMR model (Fig, **5A**). (Fig. **5B**) demonstrates the trends of exposure-response functions of the two individual chemicals. When the other chemical was at the median value, the chemical was positively linked to FAI but presented an inverse relation with SHBG. When BKMR analysis was conducted stratified by gender-puberty, more pronounced associations were observed among girls (Figs. **S1A-1D** & **S2A-2D**). PIPs are summarized in Table **S2**, MeHg was an important contributor to a positive relation with FAI (PIP=0.444), but a negative relation with SHBG (PIP=0.296).

## 4 DISCUSSION

Our study represents a pioneering effort to delve into the intricate relationship between heavy metal exposure and sex hormones in a cohort of 2,637 children, all aged 18 years or younger. Leveraging data obtained from the NHANES cycles of 2013-2014 and 2015-2016, we embarked on a quest to unravel the yet-unexplored implications of heavy metal exposure on the delicate balance of sex hormones.

It is well-established that heavy metal exposure may exert an influence on hormonal dynamics [38-43], yet research focusing on the effects of mercury exposure on the health of children and adolescents remains scarce. Univariable analysis revealed a positive association between MeHg and Hg levels and TT, FAI, and the TT/ E2 ratio, while showcasing a negative correlation with SHBG. Subsequently, multiple analyses further underscored the strong link between MeHg, Hg, and FAI, SHBG.

The function of the hypothalamic-pituitary-gonadal (HPG) axis also played a vital role in the development of human sexual characteristics [44]. As children transition into adolescence, the HPG axis orchestrates a complex process of maturation, its functionality evolving with age [45-48]. Moreover, due to the ongoing development of their organs and the limited metabolic capacity for heavy metals [49-53], children exhibit greater sensitivity to the pernicious effects of mercury. A previous study demonstrated that Hg exposure induced oxidative stress in adult gonads, leading to alterations in gonadal histology [54]. Li *et al.* discovered that exposure to metals triggered oxidative stress and provoked significant elevations in HPG-related hormones, such as corticotropin-releasing hormone and adrenocorticotropic hormone, compared to control groups [55]. Besides, Shi *et al.* also noted a positive correlation between Hg exposure and dehydroepiandrosterone levels in children [56]. These studies strongly suggested that mercury may inflict more severe damage upon the gonads of children. Furthermore, Rotter *et al.* uncovered a negative association between E2 and Hg in males over the age of 30 [57]. However, our findings demonstrate that both Hg and MeHg positively correlate with TT, FAI, and TT/E2 in children under the age of 18, implying that Hg may stimulate the production of TT and FAI through exerting adverse effects on the endocrine system.

Notably, distinctions in endocrine responses to Hg exposure have emerged between the sexes [58-61]. Sarzo *et al.* demonstrated a negative correlation between Hg and TT levels in both boys and girls [62]. Conversely, Liu *et al.* discovered that cadmium and selenium positively correlated with FAI but negatively correlated with SHBG in males, while SHBG positively correlated with cadmium and lead but FAI negatively correlated with lead in females [63]. We also observed a positive correlation between MeHg and FAI in prepubertal girls, while a negative correlation manifested in prepubertal boys. Importantly, this correlation attained statistical significance solely in prepubertal girls, suggesting gender-specific disparities in the correlation between certain sex hormones, such as FAI and SHBG, and mercury content, potentially influenced by age. Furthermore, the BKMR model unveiled the pivotal role of MeHg, positively correlating with FAI and negatively correlating with SHBG. This underscores the indispensable contribution of MeHg in the development of girls' gonads, ultimately shaping FAI levels.

Seafood consumption is a primary source of mercury exposure for children [64-67]. Maternal seafood consumption is also critical for the child’s prenatal and neonatal exposure to mercury [68-71]. Therefore, governments are committed to protecting the Marine environment with strict measures such as controlling the discharge of industrial wastewater. Since heavy metals, especially mercury released from coal and oil combustion, are transported through the atmosphere and into the ocean [72-76], efforts are also being made to establish stricter emission standards, promote clean production technologies, and strengthen pollution source supervision and enforcement.

In this study, MeHg and Hg were significantly related to FAI and SHBG in prepubertal girls, suggesting that mercury may interfere with the HPG axis or gonadal steroidogenesis during the critical developmental window period, which has important public health implications. First, public health agencies should emphasize that preadolescent girls and their families need to avoid or strictly limit the intake of fish with high MeHg content. Second, biological monitoring of blood mercury, especially FAI and SHBG for this population, can be carried out in high-risk areas of mercury pollution (industrial zones, coastal communities). Third, public education should be strengthened to enhance families’ awareness of mercury risks in seafood and their special sensitivity during the critical developmental window period (pre-puberty). These intervention measures are expected to prevent the potential endocrine-disrupting effects of mercury exposure, reduce the risk of long-term polycystic ovary syndrome and metabolic disorders, and provide key protection for susceptible populations.

For strengths, this study is based on a large-scale national survey with representatives, which helps mitigate potential bias due to the inappropriate selection of study participants. Besides, we retrieved covariates regarding previous publications and adopted multivariable GLMs to adjust covariates, enhancing the reliability of the results. Then, we focused on more sensitive groups stratified by puberty status and gender to monitor particular results. Moreover, the use of the BKMR model added novelty to this paper since it was recently developed to evaluate the effects of chemical mixtures on health. Inevitably, this study also exhibited several limitations. The participants were limited to American children, and thus our findings may not be generalizable to populations globally due to geographical, environmental, and human factors. The time of blood sampling menstrual cycle requires consideration since hormone levels fluctuate throughout the menstrual cycle; however, this index was not recorded in the NHANES. Third, hormone levels can fluctuate throughout the day, months, and across different stages of puberty, and a single snapshot may not fully capture the complexities of hormonal regulation or the developmental timing of hormonal changes; however, the database lacks hormone levels at different times. Mercury sources were not differentiated, also due to a lack of data in the NHANES database, despite known variations in toxicity and bioavailability. Besides, other important hormones such as luteinizing hormone, follicle-stimulating hormone, or progesterone are not recorded in NHANES cycles 2013-2016. Therefore, we cannot clarify whether mercury acts directly on gonadal steroidogenesis or *via* HPG axis disruption. Finally, this cross-sectional study renders a correlation rather than causality. Although our study lays the groundwork for future research into the long-term consequences of mercury exposure on sexual development and reproductive health, future longitudinal large-scale studies with repeated mercury exposure measures (including prenatal and early-life) and more comprehensive indicators (such as luteinizing hormone and follicle-stimulating hormone) are warranted to strengthen our results. Future research should be dedicated to quantifying mercury exposure from different sources more accurately. This includes using detailed dietary questionnaires (distinguishing types of seafood) and environmental monitoring data (such as pollution sources near the residence) to construct a more comprehensive exposure profile. Such research can track the temporal sequence of exposure and outcomes and more effectively control confounding factors that change over time. Besides, *in vitro* and *in vivo* experiments should be conducted to examine the effect of mercury on the activity of steroid synthase and the HPG axis signaling pathway if conditions are available in the future.

## CONCLUSION

In conclusion, our study unravels the intricate interplay between MeHg and Hg exposure and their respective associations with FAI and SHBG, underscoring the notable impact on prepubertal girls. Our findings provide a robust foundation for implementing measures aimed at mitigating the adverse effects of mercury on sex hormones in young populations, thereby safeguarding the reproductive health of children through early adoption of protective strategies. However, given the cross-sectional nature of our study, it needs to be interpreted in further studies.

## Figures and Tables

**Fig. (1):**
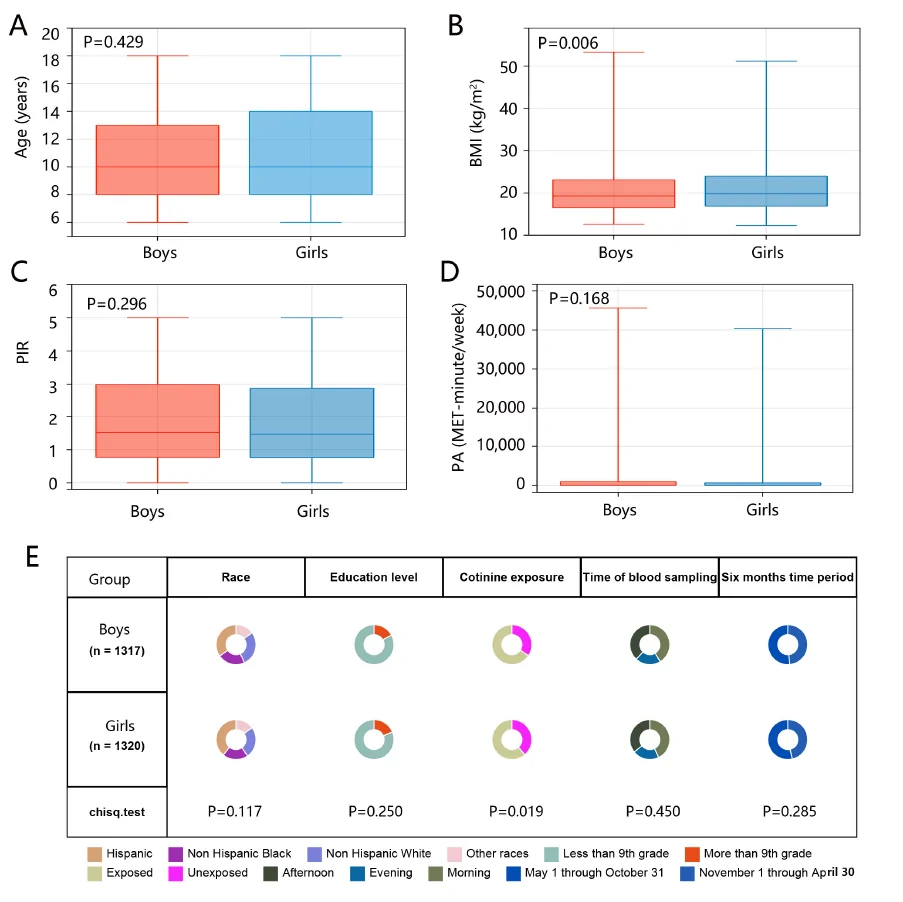
Baseline characteristics of the children stratified by gender. The continuous characteristics of **(A)** Age, **(B)** BMI, **(C)** PIR, and **(D)** PA between boys and girls. **(E)** Distribution of categorical clinical parameters. **Abbreviations:** BMI, body mass index; PIR, poverty income ratio; PA, physical activity.

**Fig. (2):**
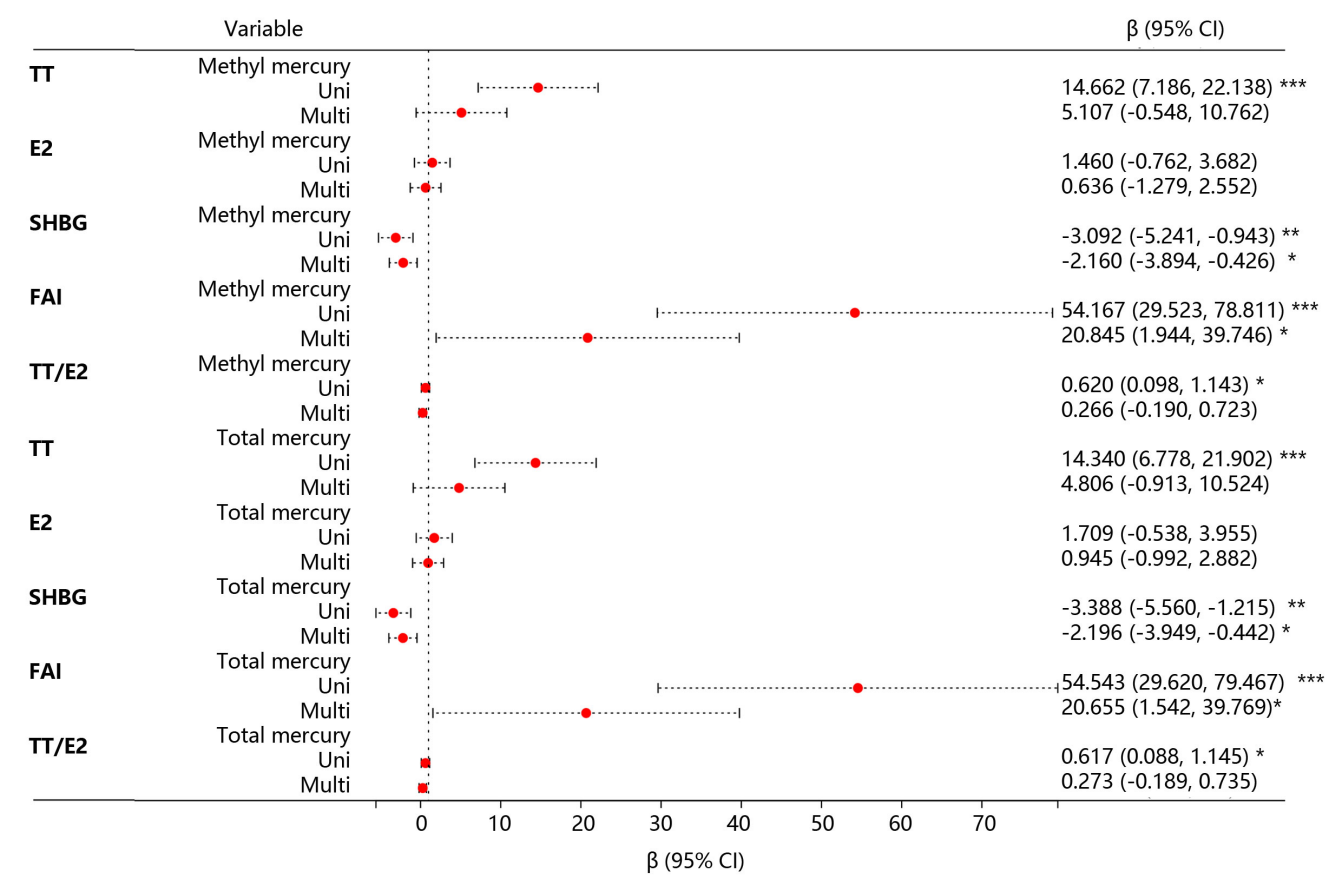
Association of single exposure as a continuous variable with sex hormone levels among all children visualized by the forest plot. **Abbreviations:** Uni, univariable generalized linear model; Multi, multivariable generalized linear model; 95% CI, 95% confidence interval; TT, total testosterone; E2, estradiol; SHBG, sex hormone–binding globulin; FAI, Free androgen index. *P <0.05, **P <0.01, ***P <0.001.

**Fig. (3):**
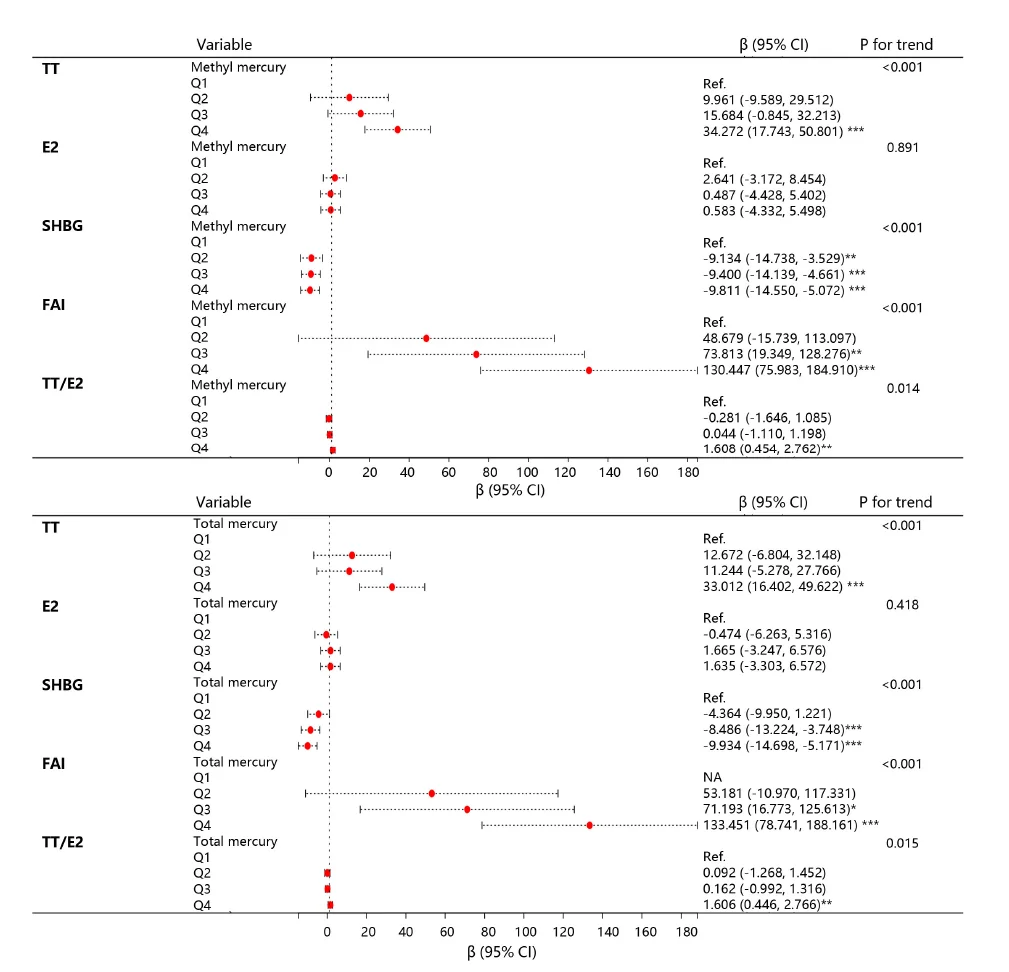
Association of single exposure as a categorical variable with sex hormone levels among all children using the univariable generalized linear model. **Abbreviations:** 95% CI, 95% confidence interval; TT, total testosterone; E2, estradiol; SHBG, sex hormone–binding globulin; FAI, Free androgen index. *P <0.05, **P <0.01, ***P <0.001. Methyl mercury quartiles: Q1 (0.08, 0.08), Q2 (0.12, 0.18), Q3 (0.19, 0.4), Q4 (0.41, 12.47); Total mercury quartiles: Q1 (0.20, 0.20), Q2 (0.28, 0.35), Q3 (0.36, 0.59), Q4 (0.60, 13.29).

**Fig. (4):**
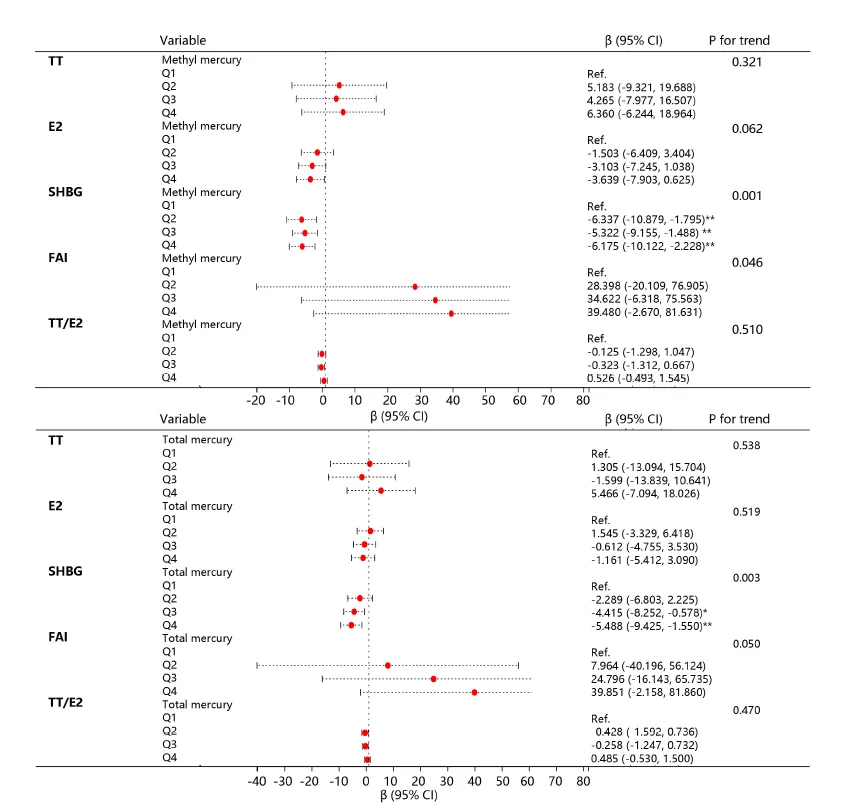
Association of single exposure as a categorical variable with sex hormone levels among all children using the multivariable generalized linear model. **Abbreviations**: 95% CI, 95% confidence interval; TT, total testosterone; E2, estradiol; SHBG, sex hormone–binding globulin; FAI, Free androgen index. *P <0.05, **P <0.01. Methyl mercury quartiles: Q1 (0.08, 0.08), Q2 (0.12, 0.18), Q3 (0.19, 0.4), Q4 (0.41, 12.47); Total mercury quartiles: Q1 (0.20, 0.20), Q2 (0.28, 0.35), Q3 (0.36, 0.59), Q4 (0.60, 13.29).

**Fig. (5):**
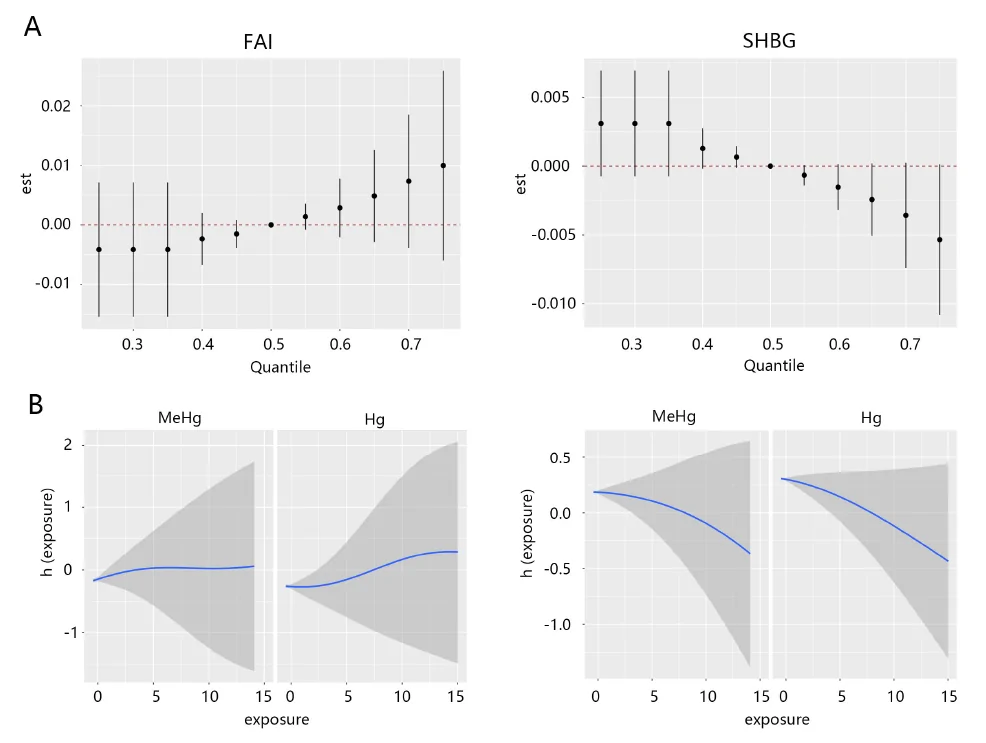
Bayesian kernel machine regression analysis. (**A**) The overall effect of the mixture on sex steroid hormones. (**B**) The exposure-response function between the individual chemical and sex steroid hormones. **Abbreviations:** FAI, Free androgen index; SHBG, sex hormone–binding globulin; MeHg, methyl mercury; Hg, total mercury.

**Table 1:** Baseline characteristics of the children stratified by gender.

Variables	Total (n=2637)	Boys (n=1317)	Girls (n=1320)	*P*-value
Age (years)	10.000 (8.000, 14.000)	10.000 (8.000, 13.000)	10.000 (8.000, 14.000)	0.429
BMI (kg/m^2^)	19.600 (16.700, 23.500)	19.300 (16.500, 23.100)	19.800 (16.900, 24.000)	0.006
Race	-	-	-	0.117
Hispanic	971 (36.822)	457 (34.700)	514 (38.939)	-
Non-Hispanic White	708 (26.849)	373 (28.322)	335 (25.379)	-
Non-Hispanic Black	569 (21.578)	293 (22.248)	276 (20.909)	-
Other races	389 (14.752)	194 (14.730)	195 (14.773)	-
Education level	-	-	-	0.250
Less than 9th grade	2165 (82.132)	1093 (82.992)	1072 (81.274)	-
More than 9th grade	471 (17.868)	224 (17.008)	247 (18.726)	-
Poverty income ratio	1.490 (0.760, 2.900)	1.520 (0.770, 2.990)	1.470 (0.760, 2.880)	0.296
Physical activity (MET-minute/week)	0.000 (0.000, 724.000)	0.000 (0.000, 960.000)	0.000 (0.000, 600.000)	0.168
Cotinine exposure	-	-	-	0.019
Unexposed	961 (36.498)	451 (34.297)	510 (38.695)	-
Exposed	1672 (63.502)	864 (65.703)	808 (61.305)	-
Time of blood sampling	-	-	-	0.450
Morning	1105 (41.904)	543 (41.230)	562 (42.576)	-
Afternoon	972 (36.860)	501 (38.041)	471 (35.682)	-
Evening	560 (21.236)	273 (20.729)	287 (21.742)	-
Six months’ time period	-	-	-	0.285
November 1 through April 30	1248 (47.327)	637 (48.368)	611 (46.288)	-
May 1 through October 31	1389 (52.673)	680 (51.632)	709 (53.712)	-
Shellfish/Fish eaten during past 30 days	-	-	-	0.205
Yes	1363 (51.688)	697 (52.923)	666 (50.455)	-
No	1274 (48.312)	620 (47.077)	654 (49.545)	-

**Abbreviations: **BMI, body mass index.

**Table 2:** Distribution of the sex hormone levels and chemical exposure levels.

Variable	LOD	Mean	Selected percentiles		
-	-	-	25th	50th	75th
**Serum sex hormone levels**	-	-	-	-	-
TT (ng/dL)	12.44	79.41	3.28	8.42	31.15
E2 (pg/mL)	22.21	23.42	2.11	3.54	24.95
SHBG (nmol/L)	52.07	76.63	38.09	66.41	106.25
FAI (TT/SHBG)	0.24	237.52	3.52	14.92	72.84
TT/E2	0.56	6.03	0.63	1.36	3.60
**Mercury levels**	-	-	-	-	-
Inorganic mercury (ug/L)	0.21	0.22	0.19	0.19	0.19
Ethyl mercury (ug/L)	0.064	0.11	0.11	0.11	0.11
Methyl mercury (ug/L)	0.26	0.44	0.08	0.18	0.40
Blood mercury, total (ug/L)	0.28	0.59	0.20	0.35	0.59

**Abbreviations:** LOD, limits of detection; TT, total testosterone; E2, estradiol; SHBG, sex hormone–binding globulin; FAI, Free androgen index.

**Table 3:** Relationship between single exposure and sex hormone levels by sex-puberty status using a multivariable generalized linear model.

Puberty status	Gender	Chemicals	TT	E2	SHBG	FAI	TT/E2
-	-	-	β (95% CI)	-	-	-	-
Pubertal	Boys (n=497)	Methyl mercury	1.659 (-13.043, 16.361)	-0.015 (-0.752, 0.722)	-0.015 (-0.752, 0.722)	39.806 (-11.948, 91.561)	0.12 (-1.290, 1.530)
-	-	Total mercury	2.083 (-12.851, 17.018)	-0.063 (-0.811, 0.685)	-1.306 (-3.435, 0.824)	47.27 (-5.256, 99.796)	0.267 (-1.165, 1.699)
-	Girls (n=587)	Methyl mercury	-0.998 (-2.222, 0.227)	1.853 (-5.307, 9.012)	1.853 (-5.307, 9.012)	1.831 (-2.511, 6.174)	-0.01 (-0.038, 0.019)
-	-	Total mercury	-0.823 (-2.016, 0.370)	2.838 (-4.127, 9.804)	-2.907 (-5.888, 0.074)	1.976 (-2.249, 6.202)	-0.013 (-0.040, 0.015)
Prepubertal	Boys (n=820)	Methyl mercury	-0.224 (-0.758, 0.309)	-0.018 (-0.062, 0.0^26^]	-2.278 (-5.366, 0.809)	-0.493 (-1.513, 0.527)	-0.104 (-0.343, 0.134)
-	-	Total mercury	-0.163 (-0.702, 0.375)	-0.022 (-0.067, 0.022)	-2.69 (-5.802, 0.422)	-0.404 (-1.433, 0.625)	-0.066 (-0.307, 0.175)
-	Girls	Methyl mercury	-0.236 (-0.633, 0.161)	0.107 (-0.272, 0.485)	-3.919 (-8.078, 0.240)	1.245 (0.075, 2.416) *	-0.039 (-0.169, 0.092)
-	(n=733)	Total mercury	-0.362 (-0.776, 0.052)	-0.051 (-0.446, 0.345)	-4.38 (-8.721, -0.039) *	0.88 (-0.344, 2.104)	-0.028 (-0.164, 0.109)

**Abbreviations:** TT, total testosterone; E2, estradiol; SHBG, sex hormone–binding globulin; FAI, Free androgen index; 95% CI, 95% confidence interval.

*P <0.05. Adjusted for age, race, body mass index, education level, physical activity, poverty income ratio, cotinine exposure, time of blood sampling, and six months’ time period.

## Data Availability

The datasets generated during and/or analyzed during the current study are available from the corresponding author on reasonable request.

## LIST OF ABBREVIATIONS

TT: Testosterone
E2: Estradiol
SHBG: Sex Hormone-Binding Globulin
MeHg: Methyl Mercury
BKMR: Bayesian Kernel Machine Regression
NHANES: National Health And Nutrition Examination Study
InHg: Inorganic Mercury
EtHg: Ethyl Mercury
Hg: Total Mercury
LOD: Lower Limit Of Detection
BMI: Body Mass Index
PIR: Poverty Income Ratio
GLM: Generalized Linear Models
PIPs: Posterior Inclusion Probability
HPG: Hypothalamic-Pituitary-Gonadal

## STANDARDS OF REPORTING

STROBE guidelines were followed in this study.
