# Supplementary material for: Associations of Mercury Exposure with Serum Sex Steroid Hormones in Children 6-18 Years from NHANES 2013-2016
Source: Endocr Metab Immune Disord Drug Targets. 2025 Sep 25;26:E18715303396515. doi: 10.2174/0118715303396515250912001734 (PMC13662729; doi:10.2174/0118715303396515250912001734)
Supplement: Supplementary file 1 [file EMIDDT-26-E18715303396515_SD1.pdf]

Supplementary Material

Associations of Mercury Exposure with Serum Sex Steroid Hormones in Children 6-18 Years from NHANES 2013-2016 : Association of Mercury with FAI and SHBG

Binwei Qiu[1,*] and Yan Lin[1]

[1]*Pediatrics, Longyan First Affiliated Hospital of Fujian Medical University, Longyan 364000, Fujian, China*

Table S1 The posterior inclusion probability of individual exposure variables to the overall mixture effect.

	**Free Androgen Index**	**Sex hormone–binding Globulin**
Methyl mercury	0.444	0.296
Total mercury	0.408	0.128

Table S2 The posterior inclusion probability of individual exposure variables to the overall mixture effect.

	Free androgen index	Sex hormone–binding globulin
Methyl mercury	0.444	0.296
Total mercury	0.408	0.128

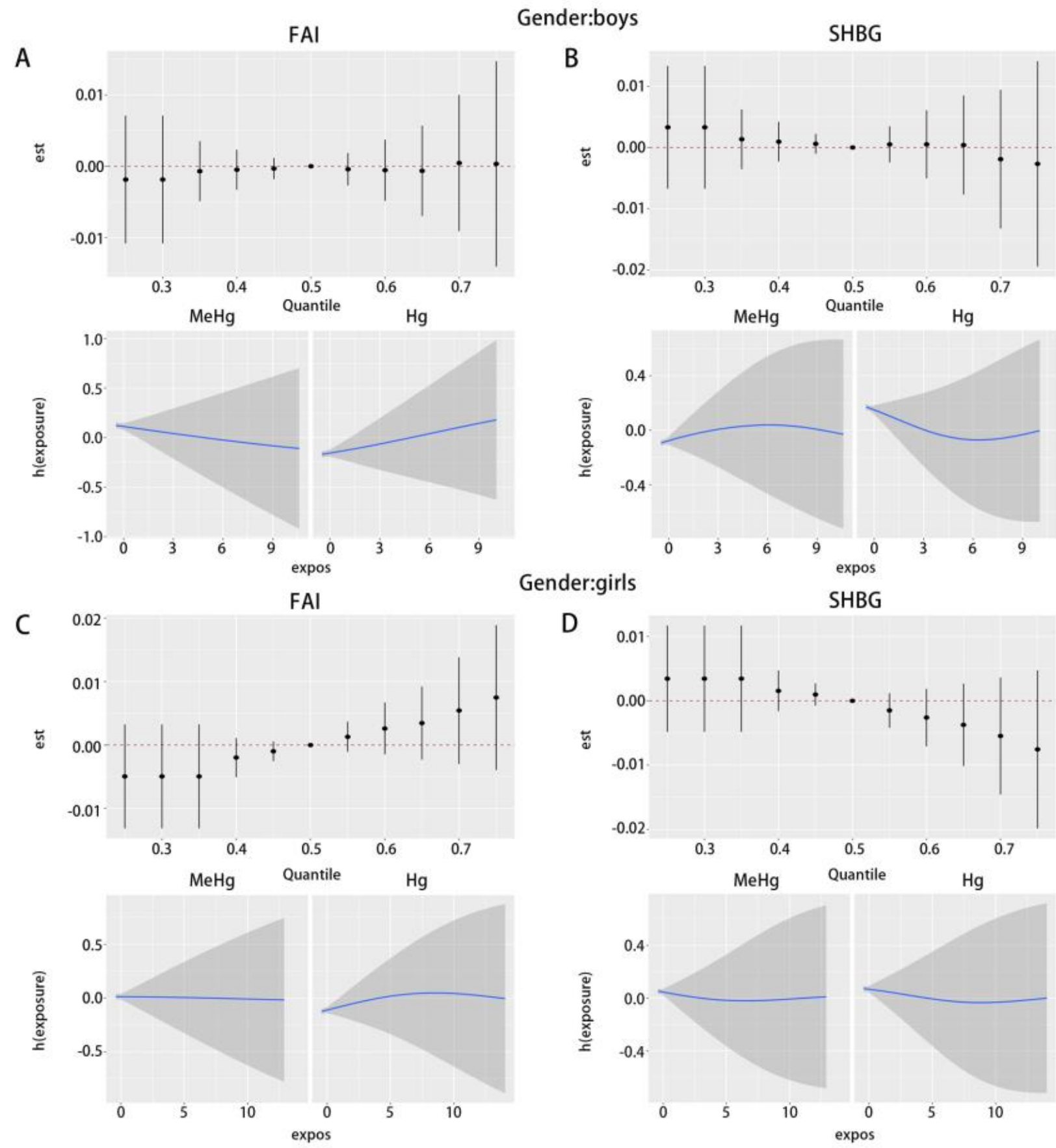

Fig. (S1). Bayesian kernel machine regression analysis in pubertal children. The overall effect of the mixture on FAI (**A**) and SHBG (**B**) among pubertal boys. The overall effect of the mixture on FAI (**C**) and SHBG (**D**) among pubertal girls. Abbreviations: FAI, Free androgen index; SHBG, sex hormone–binding globulin; MeHg, methyl mercury; Hg, total mercury.

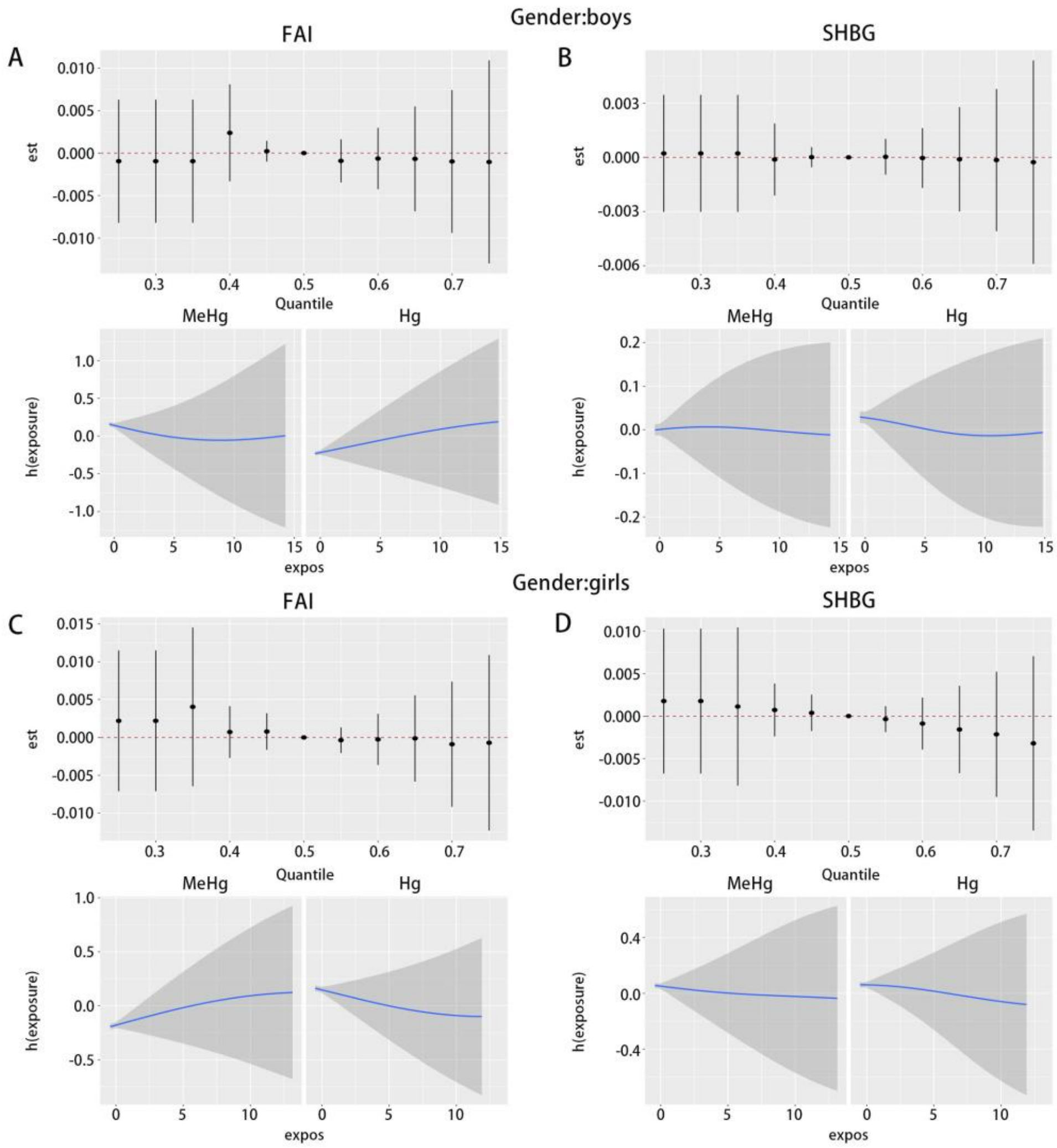

Fig. (S2). Bayesian kernel machine regression analysis in prepubertal children. The overall effect of the mixture on FAI (**A**) and SHBG (**B**) among prepubertal boys. The overall effect of the mixture on FAI (**C**) and SHBG (**D**) among prepubertal girls. Abbreviations: FAI, Free androgen index; SHBG, sex hormone–binding globulin; MeHg, methyl mercury; Hg, total mercury.
